# Characteristics of internal medicine residents who successfully match into cardiology fellowships

**DOI:** 10.1186/s12909-020-02154-w

**Published:** 2020-07-28

**Authors:** Michael W. Cullen, Kyle W. Klarich, Amy S. Oxentenko, Andrew J. Halvorsen, Thomas J. Beckman

**Affiliations:** 1 Department of Cardiovascular Medicine, Mayo Clinic, Rochester, Minnesota USA; 2 Division of Gastroenterology and Hepatology, Department of Internal Medicine, Mayo Clinic, Scottsdale, Arizona USA; 3 Internal Medicine Residency, Department of Internal Medicine, Mayo Clinic, Rochester, Minnesota USA; 4 Division of General Internal Medicine, Department of Internal Medicine, Mayo Clinic, Rochester, Minnesota USA

**Keywords:** Cardiovascular diseases, Cardiology fellowship, Career choice, Internal medicine residency, Graduate medical education, Sub-specialty fellowship

## Abstract

**Background:**

The unique traits of residents who matriculate into subspecialty fellowships are poorly understood. We sought to identify characteristics of internal medicine (IM) residents who match into cardiovascular (CV) fellowships.

**Methods:**

We conducted a retrospective cohort study of 8 classes of IM residents who matriculated into residency from 2007 to 2014. The primary outcome was successful match to a CV fellowship within 1 year of completing IM residency. Independent variables included residents’ licensing exam scores, research publications, medical school reputation, Alpha Omega Alpha (AOA) membership, declaration of intent to pursue CV in the residency application personal statement, clinical evaluation scores, mini-clinical evaluation exercise scores, in-training examination (ITE) performance, and exposure to CV during residency.

**Results:**

Of the 339 included residents (59% male; mean age 27) from 120 medical schools, 73 (22%) matched to CV fellowship. At the time of residency application, 104 (31%) had ≥1 publication, 38 (11%) declared intention to pursue CV in their residency application personal statement, and 104 (31%) were members of AOA. Prior to fellowship application, 111 (33%) completed a CV elective rotation. At the completion of residency training, 108 (32%) had ≥3 publications. In an adjusted logistic regression analysis, declaration of intention to pursue CV (OR 6.4, 99% CI 1.7–23.4; *p* < 0.001), completion of a CV elective (OR 7.3, 99% CI 2.8–19.0; *p* < 0.001), score on the CV portion of the PGY-2 ITE (OR 1.05, 99% CI 1.02–1.08; *p* < 0.001), and publication of ≥3 manuscripts (OR 4.7, 99% CI 1.1–20.5; *p* = 0.007) were positively associated with matching to a CV fellowship. Overall PGY-2 ITE score was negatively associated (OR 0.93, 99% CI 0.90–0.97; *p* < 0.001) with matching to a CV fellowship.

**Conclusions:**

Residents’ matriculation into CV fellowships was associated with declaration of CV career intent, completion of a CV elective rotation, CV medical knowledge, and research publications during residency. These findings may be useful when advising residents about pursuing careers in CV. They may also help residents understand factors associated with a successful match to a CV fellowship. The negative association between matching into CV fellowship and overall ITE score may indicate excessive subspecialty focus during IM residency.

## Background

Across graduate medical education, due to limited research on matriculation into fellowship programs, factors related to career choice and drivers of entry into subspecialty training remain poorly defined. Exposures to specific specialties during early training may impact career selection of those specialties [1, 2]. Internal medicine (IM) residents have reported that family and non-work activities influence their career decisions [3]. Surveys of IM residents have found that career intentions often develop before residency and that mentorship plays a role in subspecialty choice [4–6]. Studies have investigated potential relationships between resident achievement and career choice. A higher rank list position among general surgery residents may determine scholarly productivity and pursuit of an academic career [7]. Accomplishments during early training, such as AOA membership, scholarly output, and class rank, have been shown to predict performance at later stages of training [8–13]. However, limited evidence specifically links internal medicine resident performance, rotation experience, and career intentions with subspecialty fellowship choice.

Cardiovascular disease (CV) has been the largest subspecialty of IM for over a decade, with 2731 trainees enrolled in general CV fellowships in the United States during the 2017–2018 academic year [14, 15]. Fellowships in CV are highly competitive, with 1261 applicants for 894 positions in the United States in the 2018 appointment year [16]. Given the competitiveness of CV fellowships, understanding traits of residents who enter CV training may enhance efforts to counsel residents, improve CV program directors’ understanding of characteristics to consider when selecting fellows, and advance the literature regarding subspecialty and career choice among physicians. Therefore, our aim was to compare IM residents entering CV fellowship with other IM residents regarding (a) widely standardized measures of performance during medical school and residency, (b) expression of career intent on personal statements, and (c) exposure to CV rotations. This aim was directed at the overall goal of informing the career choice and subspecialty matriculation process for the benefit of IM residents, IM residency programs, and CV fellowships.

## Methods

### Setting and participants

We conducted a retrospective study of 8 classes of Mayo Clinic residents who matched to the categorical IM Residency Program in Rochester, Minnesota from 2007 to 2014 and completed residency in the academic years ending June, 2010 through June, 2017. We excluded residents who left the program before graduating or residents who completed the program in < 3 years.

### Primary outcome

The primary outcome was a resident matching into a CV fellowship through the Medical Specialties Matching Program (MSMP) within 1 year of completing IM residency to account for Chief Medical Residents or others who delayed fellowship match 1 year. The comparison group included all other graduating residents, reflecting those who entered non-CV fellowships or started independent practice upon completion of residency.

### Independent variables

We examined 3 categories of modifiable independent variables which reflected varying degrees of medical school and residency performance, expression of career intent, and exposure to CV rotations: (1) pre-residency variables, (2) markers of global performance during residency, and (3) characteristics of the residents’ CV experiences. For the later 2 groups, unless otherwise noted, we analyzed information available at the time of a resident’s application to the MSMP match. The MSMP match occurred in June of the PGY-2 year through the 2010–2011 academic years and in December of the PGY-3 year beginning in the 2012–2013 academic year. Therefore, information available at the time of MSMP application included data through the first half of the PGY-2 year for residents entering the IM Residency from 2007 to 2009 and through the entire PGY-2 year for residents entering IM Residency from 2010 to 2014.

#### Pre-residency characteristics

Pre-residency variables included residents’ scores on Step 1 and Step 2 Clinical Knowledge of the United States Medical Licensing Examination (USMLE), total and first author biomedical publications at the time of residency application, membership in the medical honor society Alpha Omega Alpha (AOA), and graduating medical school rank according to *US News & World Report* (USNWR) research ranking, which is a widely recognized indicator of medical school reputation [17]. We also identified if a resident declared intention to pursue a career in CV in the personal statement of their residency application.

#### Global residency performance

Markers of global residency performance included multisource evaluation scores across all rotations from the beginning of residency until ERAS opened for fellowship applications. We also examined scores on residents’ mini-clinical evaluation exercises (mini-CEX) completed prior to fellowship application as an indicator of global clinical performance [18, 19]. Evaluation scores were dichotomized as “highly professional” or not by comparing residents in the top 20% of their class vs. all other residents [20]. We included overall percentile score on the in-training examination (ITE) as a marker of medical knowledge [21, 22]. Finally, we included total number of PubMed indexed publications during residency as a reflection of academic performance, consistent with the reporting of resident scholarly activity for the Accreditation Council for Graduate Medical Education’s Accreditation Data System Annual Update. This is the only variable that encapsulated data across a subject’s entire 3 years of residency rather than through the time of fellowship application.

#### CV-specific experiences and performance

During the study period, Mayo Clinic IM residents completed CV ward rotations as PGY-1 and PGY-3 residents. Residents who entered the IM residency from 2007 through 2009 rotated through the cardiac intensive care unit as both PGY-1 and PGY-3 residents. Beginning in the 2010–2011 academic year, residents rotated through the cardiac intensive care unit once as a PGY-2 resident. Therefore, our analysis included data from 2 to 3 required CV rotations completed at the time of fellowship application for each resident in the study.

Characteristics of the residents’ CV experiences included faculty-of-resident assessments for required CV rotations, which were based on previously validated evaluations [23, 24]. We examined the choice to complete a CV elective rotation as a PGY-2 resident and the time of a resident’s first rotation in CV during their PGY-1 year, according to first versus second half of the academic year. As with overall evaluations, CV rotation evaluation scores were dichotomized as “highly professional” or not by comparing residents in the top 20% of their class versus their classmates [8, 20]. We included percentile score on the CV-specific content area of the in-training examination (ITE) as a measure of CV medical knowledge.

Study data abstracted from residency application materials were collected and managed using REDCap (Research Electronic Data Capture) electronic data tools hosted at Mayo Clinic [25]. REDCap is a secure, web-based application designed to support data capture for research studies. This study was approved by the Mayo Clinic Institutional Review Board.

### Statistical methods

Distributions of independent variables were reported as mean (standard deviation) for continuous variables, and n (%) for categorical variables. We examined relationships between independent variables and the binary primary outcome variable using logistic regression models. We examined functional form for continuous valued covariates visually using Loess plots and objectively by Hosmer & Lemeshow goodness-of-fit tests, with those deviating from the assumption of linearity in the logit categorized by logical breakpoints. Potential multicollinearity among covariates was assessed using the variance inflation factor (VIF), with the highest VIF valued covariate being excluded and re-assessing until all VIF < 3. A multivariable logistic regression model for the primary outcome adjusted for all modifiable covariates simultaneously. The threshold for statistical significance was set at α = 0.01. All analyses were performed using SAS version 9.4 (SAS Institute Inc., Cary, NC).

## Results

Over the 8 year study period, 45 residents per year matched to the categorical IM Residency Program. Of these 360 eligible residents, 20 (5.6%) did not complete the program, and 1 (0.3%) graduated after only 2 years. Therefore, our study group for analysis included 339 residents who matriculated into residency from 120 unique medical schools. Table 1 displays basic demographic data and pre-residency variables of the residents in the study and those excluded from the final analyses. Pre-residency first author and total publications (presence or absence) and USNWR medical school research ranking (top 50 or not) were dichotomized based on the functional form evaluations described above. No significant differences in demographic or pre-residency variables existed between those included versus excluded from our study.

**Table 1:** Resident demographic and pre-residency characteristics

	Eligible (***N*** = 360)^*^	Excluded (***N*** = 21)^*^	Included (***N*** = 339)^*^	***p***-value^**†**^
**Gender**				> 0.99
**Male**	211 (58.6%)	12 (57.1%)	199 (58.7%)	
**Female**	149 (41.4%)	9 (42.9%)	140 (41.3%)	
**Medical school type**				0.09
**U.S. public**	208 (57.8%)	9 (42.9%)	199 (58.7%)	
**U.S. private**	117 (32.5%)	7 (33.3%)	110 (32.5%)	
**International, except Canadian**	27 (7.5%)	4 (19.1%)	23 (6.8%)	
**Canadian**	4 (1.1%)	0 (0.0%)	4 (1.2%)	
**Osteopathic**	4 (1.1%)	1 (4.8%)	3 (0.9%)	
**Age** (years)	27.3 (2.9)	29.0 (3.5)	27.18 (2.9)	0.03
**Declared CV career intent**				0.15
**No**	322 (89.4%)	21 (100%)	301 (88.8%)	
**Yes**	38 (10.6%)	0 (0%)	38 (11.2%)	
**Top 50 medical school**				0.63
**No**	242 (67.2%)	13 (61.9%)	229 (67.6%)	
**Yes**	118 (32.8%)	8 (38.1%)	110 (32.5%)	
**AOA member**				0.14
**No**	253 (70.3%)	18 (85.7%)	235 (69.3%)	
**Yes**	107 (29.7%)	3 (14.3%)	104 (30.7%)	
**Research publication, any**				> 0.99
**No**	250 (69.4%)	15 (71.4%)	235 (69.3%)	
**Yes**	110 (30.6%)	6 (28.6%)	104 (30.7%)	
**Research publication, 1st author**				> 0.99
**No**	309 (85.8%)	18 (85.7%)	291 (85.8%)	
**Yes**	51 (14.2%)	3 (14.3%)	48 (14.2%)	
**USMLE score**				
**Step 1**	235.1 (15.9)	232.8 (17.4)	235.3 (15.9)	0.53
**Step 2 Clinical Knowledge**	246.0 (15.3)	240.6 (13.4)	246.3 (15.4)	0.07

Abbreviations: *AOA* Alpha Omega Alpha; *CV* cardiovascular; *USMLE* United States Medical Licensing Examination

^*^Data are presented as n (% of column total) for categorical variables or mean (standard deviation) for continuous variables

^†^*P*-values compare those included in vs. excluded from the study

Table 2 displays descriptive summaries of study variables, both overall and according to CV fellowship match status. Of the 339 residents in the study, 268 (79%) entered fellowship training and 73 (22% overall, 27% of residents entering fellowship training) matched to 28 unique CV fellowship programs within a year of residency graduation. Of all residents, 37 (11%) deferred fellowship match 1 year after completion of residency. The majority of these residents (60%) deferred fellowship match due to a Chief Medical Resident year.

**Table 2:** Bivariate and multivariable logistic regression analyses

		All subjects (***N*** = 339)	Matched to CV (***N*** = 73)	Logistic regression analyses			
				Bivariate		Multivariable	
Pre-residency characteristics		N (%)	N (% of)^a^	OR (99% CI)	***p***-value	OR (99% CI)	***p***-value
Research publications, total	≥1	104 (30.7%)	28 (26.9%)	1.556 (0.764–3.170)	0.11	1.166 (0.342–3.981)	0.75
	0	235 (69.3%)	45 (19.2%)	–		–	
Research publications, 1st author	≥1	48 (14.2%)	15 (31.3%)	1.826 (0.752–4.432)	0.08	1.210 (0.258–5.665)	0.75
	0	291 (85.8%)	58 (20.0%)	–		–	
Declared CV career intent	Yes	38 (11.2%)	25 (65.8%)	10.136 (3.844–26.729)	< 0.0001	6.387 (1.746–23.356)	0.0002
	No	301 (88.8%)	48 (16.0%)	–		–	
Top 50 medical school	Yes	110 (32.5%)	26 (23.6%)	1.199 (0.586–2.451)	0.51	0.891 (0.320–2.484)	0.77
	No	229 (67.6%)	47 (20.5%)	–		–	
AOA member	Yes	104 (30.7%)	24 (23.1%)	1.139 (0.550–2.359)	0.65	0.859 (0.276–2.672)	0.73
	No	235 (69.3%)	49 (20.9%)	–		–	
		**Mean (SD)**	**Mean CV vs. not**				
USMLE Step 1 score		235.3 (15.9)	237.0 vs. 234.8	1.009 (0.987–1.031)	0.29	1.027 (0.987–1.069)	0.08
USMLE Step 2 Clinical Knowledge score		246.3 (15.4)	246.6 vs. 246.2	1.001 (0.980–1.024)	0.87	0.992 (0.949–1.036)	0.62
**CV-specific experiences**		**N (%)**	**N (% of)**				
CV elective	Yes	111 (32.7%)	51 (46.0%)	7.958 (3.729–16.980)	< 0.0001	7.338 (2.839–18.969)	< 0.0001
	No	228 (67.3%)	22 (9.7%)	–		–	
Timing of first CV experience	1st half	171 (50.4%)	37 (21.6%)	1.012 (0.513–2.000)	0.96	1.116 (0.460–2.711)	0.75
	2nd half	168 (49.6%)	36 (21.4%)	–		–	
CV clinical evaluations (top 20%)	Yes	68 (20.1%)	20 (29.4%)	1.714 (0.777–3.780)	0.08	1.469 (0.414–5.219)	0.43
	No	271 (79.9%)	53 (19.6%)	–		–	
		**Mean (SD)**	**Mean CV vs. not**				
ITE PGY-2 CV percentile		62.9 (26.6)	74.6 vs. 59.7	1.025 (1.009–1.041)	< 0.0001	1.049 (1.021–1.079)	< 0.0001
**Global residency performance**		**N (%)**	**N (% of)**				
Publications during residency	≥3	108 (31.9%)	34 (31.5%)	4.069 (1.279–12.946)	0.002	4.667 (1.065–20.455)	0.007
	2	74 (21.8%)	17 (23.0%)	2.641 (0.757–9.215)	0.05	2.487 (0.557–11.100)	0.12
	1	88 (26.0%)	15 (17.1%)	1.820 (0.516–6.416)	0.22	2.523 (0.541–11.776)	0.12
	0	69 (20.4%)	7 (10.1%)	–		–	
Mini-CEX evaluations (top 20%)	Yes	68 (20.1%)	18 (26.5%)	1.414 (0.630–3.172)	0.27	2.428 (0.641–9.191)	0.09
	No	271 (79.9%)	55 (20.3%)	–		–	
Clinical evaluations (top 20%)	Yes	67 (19.8%)	17 (25.4%)	1.311 (0.578–2.977)	0.39	0.769 (0.178–3.318)	0.64
	No	272 (80.2%)	56 (20.6%)	–		–	
		**Mean (SD)**	**Mean CV vs. not**				
ITE PGY-2 overall percentile		76.5 (20.1)	75.8 vs. 76.7	0.998 (0.981–1.014)	0.72	0.932 (0.896–0.970)	< 0.0001

Abbreviations: *AOA* Alpha Omega Alpha; *CV* cardiovascular; *ITE* in-training examination; *Mini-CEX* mini-clinical evaluation exercises; USMLE, United States Medical Licensing Examination

^a^Data are presented as n (% of row total) for categorical variables or mean of those who matched vs. did not match to CV for continuous variables. Displaying the categorical variables as % of row total facilitates comparison to the overall 22% match rate into CV for the residents in this study

Almost one third of all residents, 104 (31%), had at least 1 publication at the time of residency application, including 48 (14%) with a first author publication. One third, 110 (33%), graduated from a medical school ranked in the top 50 by USNWR, and 104 (31%) were members of AOA. Of all residents in the study, 38 (11%) declared intent to pursue a CV fellowship in their residency application personal statement. Mean (SD) USMLE scores were 235 (16) on Step 1 and 246 (15) on Step 2 Clinical Knowledge. Of all residents studied, 111 (33%) completed a CV elective rotation prior to fellowship application, and 108 (32%) had ≥3 publications during residency. ITE scores were restricted to the PGY-2 year to ensure VIF < 3 for all covariates included in the multivariable logistic regression model. Among all residents, mean percentile score on the PGY-2 ITE was 77 (20) overall and 63 (27) on the CV section.

Table 2 also displays results from the bivariate and multivariable logistic regression. Multivariable logistic regression demonstrate that residents who declared intent to pursue a CV fellowship in their residency application personal statement had a 6-fold increase in the odds of matching to CV fellowship (*p* = 0.0002). Choosing a CV elective rotation as a PGY-2 showed a similar 7-fold increase in the odds of matching to a CV fellowship (*p* < 0.0001). Publishing ≥3 research manuscripts during residency was associated with a 4.5-fold increase in the odds of CV fellowship match when compared to residents with no publications (*p* = 0.007).

CV knowledge, as measured by the CV section percentile on the PGY-2 ITE, showed a positive association with the odds of CV fellowship match (*p* < 0.0001). Interestingly, PGY-2 total ITE percentile demonstrated a negative association (*p* < 0.0001) with matriculation into a CV fellowship. Overall mean PGY-2 ITE percentile was 76 vs. 77 among those who did vs. did not match to CV. In contrast, mean percentile on the CV portion of the PGY-2 ITE was 75 vs. 60 among those who did vs. did not match to CV.

Other variables in the analysis were not significantly associated with matriculation into a CV fellowship. These included publications prior to residency, medical school reputation as measured by USNWR ranking, AOA status, USMLE scores, timing of first CV rotation, and evaluation scores from required CV rotations, all residency rotations, and mini-CEX assessments.

## Discussion

This study sought to identify unique characteristics of IM residents who enter CV fellowships. We found that declaration of intention to pursue a career in CV on the residency application personal statement, choice of a CV elective, percentile score on the CV section of the PGY-2 ITE, and publications during residency were positively associated with matching into a CV fellowship. Overall performance on the PGY-2 ITE was negatively associated with matching to a CV fellowship. Other traditional markers of resident aptitude and performance, such as USMLE scores, AOA membership, medical school reputation, and clinical evaluation scores were not associated with matching to a CV fellowship.

The findings demonstrate that factors related to IM residents’ intentional choices were associated with subsequent match to a CV fellowship. These choices included declaration of intention to pursue a CV fellowship on the residency application personal statement and choice of a CV elective rotation during residency. These choices likely reflected a longstanding desire and focus to pursue a career in CV, which may be necessary for successfully matching into competitive fellowships. Our findings also support the utility of the residency application personal statement for IM residency programs despite other specialties that may place less emphasis on the personal statement [26, 27].

This study did not identify associations between variables related to clinical performance during residency and matriculation into a CV fellowship. Specifically, global and CV-rotation clinical evaluation scores and mini-CEX scores were not associated with likelihood of matching to a CV fellowship. These findings were surprising, given the competitive nature of CV fellowships. Literature also suggests that accomplishments during earlier periods of training predict subsequent performance at higher levels of training [7, 9–13, 28–30]. However, prior studies have focused on performance during or after subsequent training rather than matriculation into specific fields or training programs. Our findings suggest that, particularly among IM residents, intentional choices and motivation of the resident may supersede clinical performance as a predictor of matching to a CV fellowship. These intentional choices may include a focus on developing mentor relationships that lead to strong letters of recommendation; indeed, prior work has demonstrated that strong letters predict performance at subsequent stages of training [8]. Future efforts may explore the role of mentoring relationships prior to and during IM residency on matriculation into CV or other subspecialty fellowships.

Research productivity, unlike clinical performance, was associated with matching to a CV fellowship. Residents with ≥3 publications during training demonstrated significantly higher odds of a CV fellowship match (Table 2). This finding likely reflects the emphasis that CV fellowship programs place on academic productivity. This finding may also indicate broader mentoring opportunities for IM residents interested in CV, thus leading to higher scholarly output. Notably, about half (53%) of residents in our study who matched to CV had ≤2 publications during residency, suggesting that even modest research productivity can result in successfully matching to a CV fellowship program.

We identified a large *positive* association between higher scores on the *CV portion* of the PGY-2 ITE and likelihood of matching to a CV fellowship as well as a small *negative* association between *overall*PGY-2 ITE score and CV fellowship match (Table 2). These findings are consistent with previous research. For example, ITE scores during IM residency have been associated with medical knowledge acquisition and the likelihood of passing the American Board of Internal Medicine’s initial IM certification examination [21, 22, 31, 32]. It is likely that residents with a longstanding interest in CV devoted more time to acquiring CV-specific knowledge during residency, thus scoring higher on the CV portion of the ITE. However, an increased effort to acquire CV-specific knowledge may have occurred at the expense of general knowledge acquisition, which could reflect excessive sub-specialty focus during IM residency. The potential negative impact of an over-emphasis on CV knowledge acquisition during IM residency among residents who eventually pursued CV training may have curricular implications for both IM residency and CV fellowship programs and requires further investigation.

Studies regarding factors that impact career choice among IM residents, particularly residents that pursue a career in CV, are limited. A survey of IM residents in the United States found that work-life balance is more important among those who enter specialties other than CV [33]. Similarly, a survey of medical school graduates in the United Kingdom found that work-life balance considerations are more important among those who did not pursue CV [34]. The current work may extend these findings by demonstrating that residents who are particularly driven to pursue careers in CV articulated their intentions early in their residency personal statements, deliberately engaged in CV electives, strived for scholarly productivity during IM residency, and acquired robust CV knowledge during residency training. Taken together, our work, along with previous studies, suggests that residents pursing CV may place less emphasis on work-life balance and demonstrate a willingness to make sacrifices during training for the sake of their long-term career goals.

This study has implications for residents considering careers in CV, for residency programs, and for CV fellowship programs. IM residents considering a career in CV can use these findings to understand the common factors among their predecessors who successfully matched to a CV fellowship and adjust their behaviors to the extent necessary. Residency programs can counsel their residents interested in CV about modifiable attributes associated with a successful CV fellowship match, including pursuit of a CV elective and publication of research papers. However, both IM residents and IM residency programs must understand that many factors beyond those in this study impact the likelihood of successfully matching to a CV fellowship. Finally, CV fellowship programs can use this information to understand how residents entering their fellowships differ from other IM residents. These findings should not, however, be used in isolation to judge the quality of applicants to CV or other competitive subspecialty fellowships, as many factors beyond those captured in this study contribute to a quality application for a CV fellowship.

### Limitations

This is a single-institution study at a large academic medical center, which limits generalizability of the findings. However, Mayo Clinic IM residents in this study matriculated from 120 different medical schools and subsequently matched to 28 unique CV fellowships nationwide. While the quality of CV fellowships varies, they all remain highly competitive. Our findings should provide a universally relatable message across IM residency programs and for residents matching to CV fellowships. Furthermore, the independent variables in this study – including career intent in personal statements, USMLE scores, AOA status, mini-CEX evaluations, ITE examination scores, clinical performance evaluations, and journal publications are available to and utilized by all IM residencies in the United States. Thus, our findings are relevant to both the broader IM and CV training communities.

Residents frequently change career choice during training [35]. Our study may not have identified all residents who initially desired CV fellowships and subsequently changed their plans. Our study also did not identify an association between the timing of a resident’s first rotation in CV during their PGY-1 year and matriculation into a CV fellowship, perhaps because any CV rotation during the PGY-1 year is effectively early in training regardless of the specific month of the rotation. Future work could investigate timing of first CV exposure more broadly by incorporating exposure in medical school and later in residency. Finally, the intent of our study was to compare residents entering CV fellowships with other IM residents rather than compare residents who matched to CV with those to applied but failed to match to CV. Future work could examine predictors of a successful vs. unsuccessful match to CV. However, this would likely need to occur across multiple institutions, given the low number of residents in our program who fail to match to CV or other subspecialty fellowships.

## Conclusions

We identified that significant positive correlates of match into CV fellowships were declaration of intention to pursue a career in CV on the residency application personal statement, choice of a CV elective rotation prior to the fellowship application, publications during residency, and performance on the CV portion of the ITE. These findings may be useful for IM residents pursuing a career in CV, residency programs counseling residents who are considering careers in CV, and CV fellowship programs seeking understand unique characteristics of residents entering CV subspecialty training.

## Data Availability

The datasets generated and analyzed during this study are not publicly available to maintain confidentiality of the study subjects.

## Abbreviations

AOA: Alpha Omega Alpha
CV: Cardiovascular
IM: Internal medicine
ITE: In-training examination
USMLE: United States Medical Licensing Examination
USNWR: US News and World Report
VIF: Variable inflation factor
